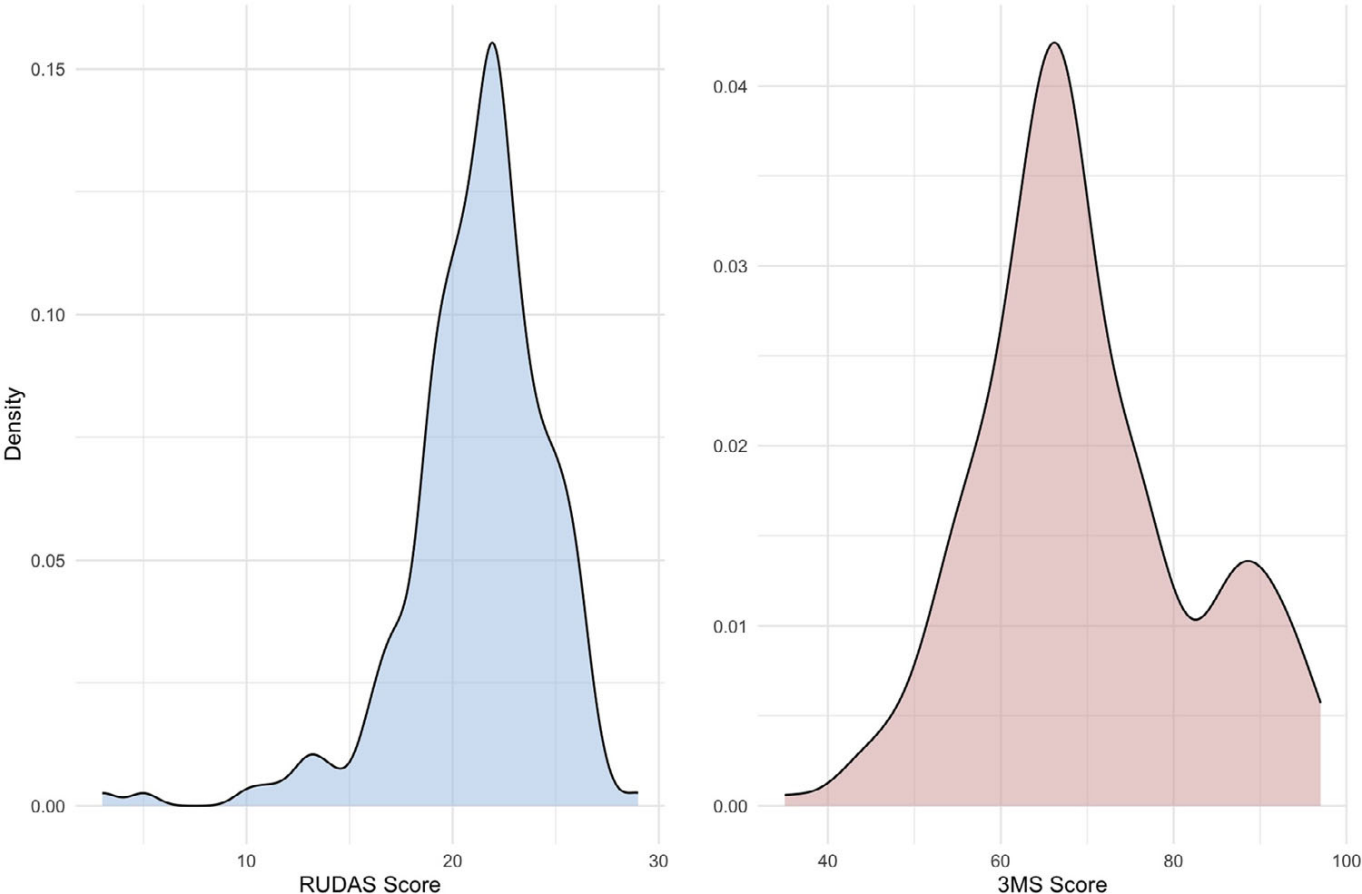# Cognitive Function Assessment in Shawi Indigenous Communities: Preliminary Data from the Peruvian Amazon

**DOI:** 10.1002/alz70860_098215

**Published:** 2025-12-23

**Authors:** Arantxa Sánchez Boluarte, Alicia Boluarte Carbajal, Danilo A Sanchez Coronel, Joseph Zunt, Hector Hugo Garcia, Monica M Diaz

**Affiliations:** ^1^ Universidad Peruana Cayetano Heredia, Lima, Lima, Peru; ^2^ School of Public Health. University of Washington, Seattle, US, WA, USA; ^3^ Universidad Cesar Vallejo, Los Olivos, Lima, Peru; ^4^ Instituto Nacional de Ciencias Neurologicas, Lima, Lima, Peru; ^5^ University of Washington, Seattle, WA, USA; ^6^ University of North Carolina at Chapel Hill School of Medicine, Chapel Hill, NC, USA

## Abstract

**Background:**

Indigenous populations in the Amazon are underserved in neurological health research, with limited data on neurocognitive disorder (NCD). Educational level, a key factor influencing cognitive function, is low in these communities. This study describes cognitive function among adults aged 50 and older in Shawi Indigenous communities of Loreto, Peru.

**Method:**

We conducted a cross‐sectional study in Shawi villages, enrolling 234 participants between November 2023 and January 2024. Cognitive function was assessed using the Peruvian version of the Rowland Universal Dementia Assessment Scale (RUDAS‐PE) and the Bolivian version of the Modified Mini‐Mental State Examination (3MS), both adapted and translated into the Shawi language. The adaptation process involved forward and backward translation, expert review, and pilot testing to ensure cultural and linguistic appropriateness. While alternative culturally‐relevant questions were developed for participants unable to answer original test items, the scores presented in this abstract reflect only responses to the original questions. Descriptive statistics summarized cognitive scores and distribution patterns.

**Result:**

The median age of participants was 58 years (56% female). Notably, 72.6% of participants did not receive formal education. The median RUDAS‐PE score was 22 (IQR: 20–23), and the median 3MS score was 67 (IQR: 62–76). Since two‐thirds of participants had never used a pencil, only 19 (8.1%) correctly drew a cube, and 26 (11.1%) were able to properly draw two intersecting pentagons. Performance in the language domain (animal‐naming task) was stronger on the RUDAS‐PE, with 79% of participants achieving a perfect score of 8. In contrast, only 11% attained a perfect score of 10 on the equivalent task in the 3MS. Preliminary analysis revealed that 80% of participants scored below previously validated cutoff scores for NCD on both tools.

**Conclusion:**

This study provides the first data on cognitive function among the Shawi. Preliminary findings suggest a high burden of NCD; however, further research is needed to refine cutoff scores and ensure accurate detection of NCD in indigenous populations by comparison with a gold standard neurological evaluation. These findings highlight the need for further adaptation of test items, particularly for populations with low literacy and different cultural understandings of memory and aging.